# Socialist Utopia in Practice: Everyday Life and Medical Authority in a Hungarian Polio Hospital

**DOI:** 10.1093/shm/hkx064

**Published:** 2017-09-14

**Authors:** Dora Vargha

**Keywords:** polio, disability, socialism, hospital, children

## Abstract

Based on oral history interviews, medical literature, hospital newsletters, memoirs and news media, this article explores the ways in which ideals of socialism interacted with medical practice in polio care in 1950s Hungary. Through the everyday life of polio hospitals, it argues that the specific care that polio demanded from hospital staff, parents and children, resonated with state socialist political discourses of gender equality and the breakdown of class barriers and conventional hierarchies in medicine. Providing opportunities, as much as failing to fulfil expectations of patients, parents and medical staff, polio care simultaneously created socialist utopias and demonstrated the limits of political ideals.

‘One day a mother brought her child to our outpatient service. The child had a high fever and we both knew what was happening. I took the child in my arms and started running, shouting for the doctor. I could feel her body go limp in my hands as I was running. By the time I reached the end of the corridor, she was completely paralyzed.’^1^The onset of polio was always a dramatic process, for patients, children and health workers alike. The Hungarian physical therapist, for whom this experience from the 1950s was so formative that it brought tears into her eyes as she recounted the story some 50 years later was not usually responsible for treating acute cases of polio. Nor was the institution this happened at an infectious disease hospital, where children who contracted the disease would initially be treated. But polio had a way of transgressing boundaries and turning over hierarchies as epidemic outbreaks raged throughout the 1950s in Hungary. The Heine-Medin hospital, a national centre of restorative care for polio patients in Hungary that was established along with other wards and institutions prompted by the growing numbers of polio patients, was no conventional hospital—just as polio was no conventional disease.

Polio care in a radically changing society, against the dramatic backdrop of a war-torn and impoverished environment, contested power relations within and without the medical profession. The disease and its treatment directly engaged with the political landscape, and the promises and failures of the state. This experience of polio behind the Iron Curtain can best be understood through a closer look at life in polio hospitals in Hungary during socialism. Children with polio often spent years in these institutions. The hospitals were sites of treatment, medical and technological innovation, and also served as sites of everyday life: a school, a home away from home, and its staff as family away from family.

Poliomyelitis, which has been known as infantile paralysis or Heine-Medin’s disease in Hungary is an infectious disease caused by a virus. It attacks mainly children and has the potential to cause permanent paralysis primarily in the limbs, but can also affect respiratory muscles or the whole body. It was a relatively new disease in the twentieth century, with its mode of transmission debated well into the 1950s, when severe outbreaks started appearing across the globe.^2^ In 1955 Jonas Salk developed a killed virus vaccine, and other researchers such as Albert Sabin, Hilary Koprowski and Herald Cox worked on oral, live polio vaccines. While the polio vaccines were heralded as symbols of medical progress in the golden age of therapeutics, their efficacy and safety were hotly disputed throughout the decade.^3^

Polio treatment was equally debated in the era. A therapy developed by Australian nurse Sister Elizabeth Kenny revolutionised polio care across the globe by warming and exercising the muscles of patients in the acute phase of polio, instead of immobilising them, which was the standard procedure until that time. Kenny’s treatment was surrounded by controversies and met with stark resistance from much of the medical establishment.^4^ Although in a very different political and health care structure, Kenny’s story exposed ways in which polio care contested medical and gender hierarchy that were similar to the socialist context.

Historians, such as Naomi Rogers in the United States, Rosa Ballester and Maria-Isabel Porras Gallo in Spain, or Dilene Raimundo do Nascimento in Brazil, have demonstrated the ways in which political ideologies and relationships between state and citizens have shaped the experience of polio and its medical treatment in various societies.^5^ While the national, and indeed global epidemic crises of polio took place in the unfolding Cold War that divided the world between East and West, a socialist perspective has been strikingly missing from the narrative of polio. A closer look at how polio treatment played out in a communist context highlights how local politics of medical care, formed along geopolitical divisions, shaped the experience of a global epidemic for medical professionals and patients alike. Furthermore, through the lens of polio we can gain a more nuanced understanding about the expectations, ideals and realities of a nascent communist society.^6^

Polio had been recorded in Hungary from the end of the nineteenth century and epidemics had been reported beginning in 1912.^7^ However, never before did the disease receive so much attention and concern from authorities, and never had the number of victims been as high as in the 1950s.^8^ Outbreaks became more severe every year, causing recurring fear summer after summer until the early 1960s. As opposed to trends in the demographics of polio epidemics in the United States, where the age groups affected by the disease shifted to older children and adults, polio in this part of the world attacked younger and younger populations as time went by.^9^ The hospitals, therefore, were filled with children ranging from a few months to school age, who often spent most of their time in these institutions for years, if not decades.

The era when polio outbreaks hit Hungary was quite out of the ordinary. Recovering from the enormous losses of the Second World War in terms of population, wealth and infrastructure, Hungary embarked on a state-building and ideological project in the 1950s: communism. Part of the forming Eastern Bloc, under Soviet influence, communist (or socialist, a term which was used interchangeably in the era) regimes governed Hungary’s foreign and domestic politics, economy, and social and cultural life for 40 years from 1949.

As many historians of Eastern Europe have pointed out, the ‘communist takeover’ was neither a sudden and complete transformation, nor was the system as totalitarian as previously believed.^10^ Political instability and social transition marked the decade: the 1950s saw the rise of a Stalinist dictatorship, political reforms after Stalin’s death, and a bloody revolution in 1956 with its aftermath and retributions that lasted until the early 1960s. Throughout the decade political ideologies and practices were enforced, abandoned, negotiated and resisted as regimes changed every couple of years.

The extraordinary times of the 1950s and the nascent political system intersected in various ways with the new epidemic challenge of polio and the disease’s treatment regimen. The treatment of polio involved factors that major targets of communist reforms. Women, playing crucial roles as physical therapists and nurses in the hospitals, were drawn into the labour market and, at the level of rhetoric, at least, were proclaimed equal to men. Children, the objects of care were also central to the new political project. Shaping the minds and bodies of children was seen as crucial in establishing a bright, socialist future.^11^ Finally, on the geopolitical scale, access to health care and overall reform of public health was a key idea that set the East apart from the capitalist West and was seen as fundamental to a successful socialist society.^12^

While most of these reforms failed in society as a whole, polio and its specific treatment created a microcosm in polio hospitals where we see the ways in which medical professionals, technical staff, children and parents drew on socialist arguments, ideals and rhetoric to pursue their professional and personal aims. Most of these processes did not emerge from ideological conviction; in fact, many took place in spite of the overall social, political and cultural context. Rather, what we see is the particularities of polio care mapping onto the particularities of a nascent socialist regime.

## The Hospital as Socialist Utopia

Following a diagnosis of polio (marked by the onset of paralysis), most patients were transferred to the national centre for infectious diseases, the László hospital in Budapest, which was also home to the largest iron lung ward in Hungary until 1959.^13^ Further polio centres were established in Debrecen and Miskolc, both in the Eastern part of the country, in counties where polio epidemics hit the hardest. From 1957, a child could be transported to one of the three iron lung wards in the country by airplane.^14^ It was quite arbitrary, however, who was directed to which infectious disease hospital. As an article in the public health journal *Népegészségügy* pointed out in 1957 ‘there [was] no directive what kind of infectious disease patients can be admitted to which hospitals under what circumstances and neither infectious disease hospitals have prescribed districts to operate within’.^15^ The significance of which infectious disease hospital a patient was admitted to was, apart from the varying quality of equipment and staff, that it usually determined the course that the patient’s polio care would take. For instance, most of László hospital’s patients were directed to the Heine-Medin hospital.

Acute treatment could be very uneven across space and time in the first days and weeks after the patient was diagnosed and admitted. In one hospital, a toddler in 1954 could be exposed to X-ray treatment and regular lumbar punctures, an extremely painful procedure when administered into an already inflamed nervous system.^16^ However, in another institution in 1959 a child of similar age could get state of the art treatment as pioneered by nurse Sister Kenny from across the globe: wrapping the muscles in warm bandages and hot packs to retain their flexibility.^17^ At the same time, many children in Budapest could receive medical attention instantaneously and have a physical therapist come to their home for regular sessions.^18^ Just as polio could have different manifestations in patients, causing a wide range of degrees of disability, the clinical care of the disease reflected this variety. Part of this was due to the diversity inherent in the disease itself. Another was due to the fact that there were no entrenched or established practices in place at the time, and treatment methods were continuously debated at international conferences throughout the decade.^19^ In a country recuperating from war with its society in turmoil, clinical practice depended to a large extent on the access of physicians to information and equipment, and of parents to the economic means to seek out the best possible institutions.

In response to the quickly escalating public health issue, polio wards in rehabilitation institutes and hospitals began to open in the 1950s. The centre of restorative care was in the capital, Budapest, where patients were divided across several institutions. The oldest of them was the Home for Crippled Children established at the turn of the century, where little medical care was taking place.^20^ With the growing number of polio patients, the National Rheumatology and Physiotherapy Institute, established in 1951, opened a new ward for polio patients in 1954. This institution was connected to a thermal bath and placed balneotherapy as a central element of treatment. Finally a hospital exclusively specialised on the disease, the Heine-Medin hospital opened in late 1956.^21^ The treatment, hospital stay and equipment (leg braces, respiratory devices, shoes, etc.) were provided by the state without charge to the patients and their families. This was a crucial point in how the socialist state imagined itself and the public health system it aimed to build. Hospitals operated with meagre resources, but offered long-term stays: some children lived a good part of the year in these institutions for up to a decade.^22^ Some parents were able to move across the country to be close to their child being treated in a specialised hospital in Budapest; they rented a room or flat near the hospital and looked for new work opportunities around the area.^23^ However, most parents were not able to uproot their whole families and find work near the treatment centre, and thus they were not often able to visit their sick children.

Polio hospitals and wards in Hungary, such as the Heine-Medin hospital could be understood as total institutions, a concept put forward by sociologist Erving Goffman that very much resonates with ideas of totalitarianism.^24^ A total institution in a ‘totalitarian’ communist state would be an unsurprising and straightforward framework of analysis in a hospital secluded from the rest of society, where young patients spent a considerable amount of time. However, when analysed from the perspective of everyday life, the picture of patient experiences and medical hierarchies becomes more complicated and does not necessarily conform to either model.

First, it was not only patients who were controlled. The unique features of polio (its permanently debilitating effect, combination of surgeries and physical therapy, plus long hospital stays) and the special care young patients required contributed to the peculiarities of the inner life of polio-treatment institutions and to medical decision-making processes. A high level of cooperation was needed for the treatment and caretaking of the disabled children, who were often very young, in pain, separated from their families and required continuous and attentive care.

The treatment of the disease and the needs of children required a large staff in the hospitals. While the children’s lives were controlled by the staff, the everyday lives of the staff were in turn controlled by the requirements of the treatment and care of the polio-stricken children. Staff working at the iron lung ward were perhaps under the strictest control, since the machines, as well as the care for children with respiratory paralysis, needed to operate like clockwork.^25^

The Heine-Medin hospital, secluded among the hills of Budapest is an ideal environment to study the ways in which polio care, medical work and ideology mutually shaped each other. Lead by a charismatic director, the hospital staff embodied a number of features of a utopian socialist society that jumbled traditional medical and social hierarchies. This might not have been an explicit goal of Dr László Lukács, whose remarks in his memoir reveal that he valued religious and anti-marxist views.^26^ An emphasis on traditional values would not be uncommon coming from a leading doctor in the 1950s.^27^ The medical professionals at the height of their careers in the 1950s and 1960s, especially the men, had been educated in the conservative interwar era.^28^ At that time the majority of medical students came from bourgeois families, which were the basis of the right wing government’s support.^29^

This micro society subordinated its life to a higher ideal, an altruist cause: helping sick children and repairing disabled bodies. All medical staff, ranging from the director to assistant nurses needed to be up-to-date with the exact medical history of each child under their care, and also needed to be ready to perform everyday tasks such as nappy changing, carrying children around on the winding staircases of the hospital, and to working regular overtime shifts.^30^ A proportion of the nursing staff lived on the premises, while other staff members would bring their own, healthy children to work if they did not have a family member available to babysit them after daycare or school.^31^

Members of the staff, in exchange for the exhausting workload and strict environment, received numerous social benefits. The result of this was that the hospital became a site for the social and family life of the staff. They received five daily meals; staff were granted extra days off and were encouraged financially to pursue higher education studies; excursions, summer holidays and parties were organised on a regular basis for the workers of the hospital; a nurse dormitory was set up in one of the buildings, welcoming nurses with financial or housing troubles.

In this way, the hospital did conform in certain ways to a concept of totality, which included staff and patients alike. Moreover, the utopian society of the polio hospital also had features that we readily associate with the ways in which totalitarian societies work: as the therapy required instructions to be followed very strictly, the director often held surprise inspections of his staff and patients. Moreover, Lukács, adhering to practices of stereotypical communist leaders, confessed to the head physiotherapist at one point: ‘I have informants among you, because I need to know about everything.’^32^

This authority and the hierarchical structure was not uncontested, however. Some members of staff utilised the peculiar hierarchy of the hospital, or lack thereof, to challenge traditional features of Hungarian society. One of the recurring themes in the internal newspaper for staff, the *Heine Medin Herald*, was the debate on workers addressing each other at the workplace. There was an effort from the editors to encourage the use of modern communist phrases like ‘comrade’ and ‘colleague’ instead of traditional pre-war phrases, like ‘sir’. It was this debate that one of the male workers joined to get his voice heard: a driver, who occupied a job that had relatively low status. Traditional social values and modern, communist ideas conflicted in the driver’s petition for equal treatment. He protested against doctors and nurses calling him by his first name and insisted on a respectable mode of address for himself.^33^

## Children in Medical Decision-making

The nature of polio treatment scrambled customary hierarchies in polio hospitals, in terms of medical authority as well. In the focus of care were the children, who, in the wake of a terrible war, were key to laying the foundations of a new society and to the political project. Polio threatened these tokens of a future healthy and productive society, and while epidemics were raging in the country, their care and the rehabilitation of their bodies became a priority. Hospitals, operating in the 1950s as an arm of the state, and at the same time as autonomous spaces, in turn became a terrain on which the role of children and authority over their care was played out.

The web of family and professionals contributing to the children’s treatment and each group’s investment and concept of the patients’ health and healing required constant negotiation from all sides. Authority over the child’s body was often intensively disputed. One of the patients remembers how the surgeon started shouting with her parents when he found out that they were gathering information about alternative methods of care. She ended up having the most common surgery in the Heine Medin hospital: to correct the uneven length of her legs and ease walking. The hospital director, Dr Lukács sawed off a part of her ‘normal’ leg near the knee joint. ‘Instead of one bad and one good leg, I ended up with two short ones. This was [the doctor’s] specialty, he liked butchering. I still remember the pain and the sound of him tinkering away at my bones. You see, they no longer used ether at that time, and even though they said I would not feel anything, I can tell you that I felt every single thing.’^34^ Erzsébet never forgave the doctors for this surgery and lamented that her parents were not in a position to protect her from such invasive operation and stand up for an alternative treatment.

In certain cases, however, the wishes of the patient and his or her parents opened new and innovative ways in otherwise routine procedures. Pál Kelemen, an 11-year-old boy and already a gifted cello player needed surgery to secure his dislocated hip. The usual method of treating this common problem was to ossify the joint, creating a sturdy but unflexible hip for the patient. However, Pál and his parents (a peasant and a factory worker) viewed this as an unworkable option, because the procedure would have rendered the boy unable to sit down to play the cello. They worked together with the surgeon to find a different method that would allow their child to continue his musical career. They even managed to book an appointment at a Viennese clinic (no small feat, as it required connections across the Iron Curtain). In all the negotiations, Pál was an active participant. ’Of course I was part of the whole process. I was in a conscious age by that time and it was my body and my life in question.’^35^ In the end, the surgeon came up with a complicated but feasible option in 1959. ‘Director Lukács performed a huge surgery on him. He was arranging Pál’s bones and muscles for half a day in the operating room’ the internal newsletter of the hospital recounted: the operation was unusual and important enough to let all staff and patients know about it in the monthly publication.^36^ The child’s resistance to the usual medical procedure and his parents’ investment in seeking alternative solutions eventually paid off. Pál grew up to be a renowned musician and became a distinguished member of the Franz Liszt Chamber Orchestra, giving concerts all over the world, including at Carnegie Hall and the Sydney Opera House.

If we look closely at individual cases, it becomes clear that there was no typical form of treatment. This was partially because the effects of polio varied significantly, ranging anywhere from a mild and mostly reversible paralysis of one limb to the complete paralysis of the body, leaving little more than facial muscles under the patient’s control. But equally important in the way treatment was structured were power relations among the groups working together and negotiating knowledge, practice and objective in the process.^37^

Thus it is very important to investigate the agency of the various actors in the power relations of the disease, including that of children.^38^ Although their histories are often difficult to trace, examining children’s experiences and their stories in their own terms is crucial in understanding medical practices, disease management and societies in general. Moving away from seeing children merely as subjects of governance and focusing on how polio patients engaged with their own medical care, we can see disabled children, often viewed as especially powerless in a hospital context, contesting and negotiating or accepting their treatment, and the techniques through which they were excluded from, or involved in, medical decision making.^39^ Moreover, children have been central to how a health society has been imagined and pursued, and this was particularly true for state socialist Eastern Europe.^40^ How their assigned political importance manifested (and the ways it did not) in medical care can help us understand not only the subtle differences of medical practice in different societies, but can offer a new insight to socialist societies in general.

In the story of polio in Hungary, children were active participants and subjects, rather than objects of care. Children’s power in shaping their own treatment had serious limitations, as parents or medical staff could overrule and coerce treatment options on them at any point. Nevertheless, it would be a mistake to dismiss children’s agency in medical care, especially since their participation was often recognised in medical practice and theory.

‘It was the time of the grand rounds. The head of the ward, the chief doctor, medical students, everyone, went from bed to bed. When they reached mine, they said I would need yet another surgery. By that time, I had been through a couple and knew exactly what that meant. So I started screaming and put all curse words I knew to use. I had been sharing a hospital room with adult men, so believe me, I knew quite a few. I sent the whole company to hell. They must have been taken aback, for the whole group quickly ushered out of the room without a word. I was 8 years old then.^41^ After the boy successfully scared off the whole medical staff, they sent a diplomatic envoy, his favourite physical therapist, to talk to him. She managed to calm him down and persuaded him to agree to a meeting with the surgeon before he made his mind up about surgery. After a discussion with the surgeon he eventually agreed to the operation, which was not to be his last.

The story points out that children’s opinions and willing participation in their own treatment was seen as crucial in polio care, even if it is difficult to say how representative the above example is. In a journal article from 1963, Dr Mária Tarnóczy, a leading rehabilitation therapist, wrote that restorative care ‘has to be considered on an individual basis, only then—and with exceptional patience in our work—can we reach satisfactory results. Consensus and cooperation between doctor and patient is nowhere else as important as in the rehabilitation of paralysis’.^42^ Since treatment involved long hours of often painful or boring physical therapy sessions, spread out over months or years, therapists thought it to be essential that children agree to their treatment and participate in it willingly. Although the surgery would probably have been performed on the child quoted above, whatever his opinion was, it was essential that he agreed with his treatment.

This approval also fit into the perception of the psychological treatment of the patients, which was seen as crucial for producing a fully valued member of society, someone who would not lose contact with the outside world, would be able to participate and interact in society, and would engage in some kind of productive work. As the work of historians such as Amy Fairchild, Daniel Wilson and David Serlin has shown, children’s restorative care in general, and polio in particular, drew on ideals that often signified priorities in a given society: masculinity, individual willpower, production, military victory.^43^ In the Hungarian case, it was work that treatment was built around. The communist project was one based on the work of all members of society, most importantly physical work. Striving for ideal, or at least ‘normal’ bodies was the cornerstone of the treatment, in the context of murals, statues and propaganda material that inundated all sites of everyday life in the 1950s. The success of polio treatment, the creation of a particular, work-oriented physical ability that would enable participation in the communist project, depended greatly on the willing participation of children in their own treatment. Therefore the hospital setting strived to create an interior world that mapped the outside one. For this reason, the Heine-Medin Post Treatment Hospital broadcast regular radio programmes for the children, operated a small zoo in the garden, and was equipped with a library.^44^ Another institute in Budapest with a large polio ward, the National Rheumatic and Physiotherapy Institute organised plays with the participation of the children.^45^

Treating children on the emotional level, not merely the physical level, was important to the perception of polio care. Every stage of the treatment, from preparing for surgeries to preparing for the outside world, had to be conducted with psychological effects in mind. ‘[The task is] on the one hand, the normalization of the psychologically damaged patient due to his/her disability. The main point here is to help adaptation to a healthy environment. On the other hand, taking the mobility of the individual patient into account, to conduct examinations for working ability and spotting potential talent.’^46^

Adaptation to a so-called healthy environment, meaning one populated by able bodies, did not necessarily mean that children were to be exposed too much to their healthy peers. While children were considered to be partners in many ways in their polio treatment and often could intervene in, or shape the management of their care, they were also seen as emotionally vulnerable because of their physical condition. This stance also signalled that there was not much space imagined for disabled children in the communist society as a whole.

## Physical Therapists: Women in the Hospital and Communist Society

The role of the physical therapist as a facilitator and negotiator in the story recounted here highlights the importance of the professional in establishing trust, forging a close relationship with the children, and providing the majority of the treatment. It was the physical therapist who would help to ‘re-tone’ muscles after weeks in a cast following surgery, and supervise practice using the modified limb. Physical therapy sessions were central to polio treatment, and life in the hospitals was organised around them. As a scientific journal article on the pressing issues of polio care claimed, ‘Among the latest treatment procedures, physical therapy has a significant importance in the rehabilitation phase. In this process, it is important not only to treat the paralysed muscles and to develop coordinated movement, but also to condition healthy muscles so that static balance can be achieved.’^47^While surgery would enable children to move their paralysed body parts to a certain degree, physical therapy’s aim was the capability for movement for the whole body. It was not merely a tool of rehabilitation after surgery, but a preparation for a future, mobile life.

Although physical therapists were central to polio care, their training on a national, organised level was non-existent in Hungary until the mid-1950s. The two-year education programme for physical therapy, offered in Budapest, came into existence because of the high demand. By the late 1950s, the escalating epidemic waves flooded hospitals with an ever-growing number of patients in need of therapy. In 1957, the year of the most severe epidemic, two more schools opened in Debrecen and Miskolc, and by 1959, more than 200 physical therapists were active.^48^

In fact, the staff of the Heine-Medin Hospital trained many of these physical therapists. The institution started out with 12 therapists, who joined Dr Lukács from the Bókay Children’s Hospital and András Pető’s institute (which developed Conductive Education, now practised worldwide).^49^ The physical therapists worked from 8 am to 6 pm daily. Each building had a leading therapist, who was supervised by the head therapist of the hospital, Judith Enyedi. Physical therapists regularly filled out time sheets containing the details of their work. ‘The treatment for all began with stimulation, moving all limbs thoroughly. Then came the individual treatment: warm packs, bath, underwater massage (tangentor), electrostimulation, casts as needed.’^50^ These therapists were not initially specialists in polio. In fact, nobody was. Developing their knowledge through practice, the core therapists became nodes of knowledge and held training sessions in several hospitals in Budapest and across the country.^51^

Polio’s significance in the professional development of physical therapists and the field itself was not unique to Hungary or to communist states. Apart from war, polio epidemics creating large numbers of children in need of rehabilitation across the globe challenged physical therapists and often provided them with opportunities in various societies from Brazil to the United States and Spain.^52^

Even though physical therapists didn’t hold a medical degree, they ranked above nurses in the professional hierarchy, due to the vital nature of their work. That work also allowed them to develop close working relationships with physicians, nurses and the children themselves. They were the ones who took part in the treatment process most intensively, having daily sessions with patients, observing surgeries, consulting with the surgeons and being companions and friends to nurses.^53^

Since the keystones of successful rehabilitation were the long, tedious and often painful processes of physical therapy and a need to follow specific exercise routines, the expertise of physical therapists gained special importance and affected their status in the medical hierarchy. Physical therapists, almost exclusively women, could overrule male surgeons in questions of polio care.^54^ ‘I told the chief director that this child is under my care and I will not allow him to operate on her. Only when I am through, and there is still a reason to operate, can he touch the patient.’^55^In treatment, physical therapists worked together with and also offered an alternative to surgeons. Some children ended up not going under any surgery at all. One patient’s mother, who worked as a physical therapist, forbade all invasive treatment and gave her daughter private physical therapy sessions after work hours.^56^

This inversion of hierarchy in medical decision making and the possibility of overruling the hospital director in questions of treatment show quite distinct gender dynamics from those usually discussed in relation to physical therapy and the medical profession. In Hungary, physicians trained physical therapists initially, moreover, it was doctors who established their field of expertise. However, this did not prevent physical therapists gaining a certain autonomy and an equal footing on certain questions of treatment in polio care.

Physical therapy in Hungarian polio care was at once the product of the medical profession in response to an epidemic challenge, and was also the product of a particular political system. Since the space and staff required for treatment in socialist countries, such as Hungary, was provided by the state under universal, free health care, children spent very long stretches of time in these institutions, and thus physical therapists, physicians and nurses worked alongside each other for years with each patient on a daily basis. Therefore, the various people performing medical care in polio wards and hospitals were the members of the same institutional unit, and were also united through clearly articulated common goals of physical rehabilitation.

Moreover, women in 1950s Hungary could draw on a discourse that favoured strong and powerful women. From female partisans to the mythical figure of tractor-driving women, physical therapists were surrounded by role models put forth in state propaganda by a regime that—at least theoretically—championed gender equality. While women’s historians have pointed out the double (or triple) burden of women in socialism and the many ways in which women were pushed back into traditional roles, recent scholarship has been exploring the multiple ways in which women were not only affected, but also responded to policies regarding gender.^57^ As the case of female physical therapists carving out new professional roles in response to a health crisis demonstrates, women occasionally did manage to successfully negotiate new roles in a new society in which political discourse proclaimed gender equality.

Of course, tension between political rhetoric and experiences on the ground existed and this was not lost on contemporary women, who sometimes gave voice to their disgruntlement. On the pages of one of the major national newspapers from International Women’s day in 1957, a female paediatrician explicitly addressed what she called the ‘second shift (after work, at home)’. She complained in the name of all women that although by law both sexes were now equal, this was far from reality, mostly due to the expectations of men and the ‘generosity’ of women to comply with men’s requests. Her solution was to raise the next generation in such a way that this would not be a problem for women in the future.^58^ Some women challenged the double burden in individual ways: Pósa Dezsőné, a former nurse at László hospital’s respiratory ward, divorced her engineer husband, who had a hard time accepting her workload and neglecting her duties as a wife: she would take night shifts when she was not on duty or would not leave the hospital immediately when her shift was over.^59^

## Living with Machines

The access to continuous care in the framework of the socialist health care system paired with a lack of resources created a community in the respiratory ward that challenged concepts of childhood, families and conventions about medical knowledge and caretaker roles. Medical staff assumed parental duties, nurses worked with highly specialised medical knowledge and also doubled as technicians, and children became active participants in shaping their own treatment.

Since the new respiratory wards, just like the other buildings of the hospital, were not originally intended to house the respiratory machines and patients, the distribution of children and devices gained a unique pattern. The amount of time children spent on the respiratory ward varied. Depending on the level of paralysis and the type of polio contracted, some spent a lifetime within the walls of the villa, while others returned to the main buildings or home as they became independent of the machines. Out of the 90 polio patients treated in the respiratory ward, 15 were unable to spend any time without mechanical ventilation. ‘In their case, the fight was for being able to spend 30 minutes outside the respirator’, wrote the head of the ward, Dr Kiss Ákosné, in her dissertation on the life of respiratory patients treated in the Heine Medin Hospital.^60^

It was the respiratory patients who spent the most time in the institution. The majority of the patients with the most severe cases of respiratory paralysis never went home after entering the hospital. György Kárpáti, for instance, contracted polio in 1959 at the age of 3 and he lived in the same iron lung in the villa until 2006, when his over 60-year-old machine was exchanged for a modern respiratory device.^61^ The prolonged stays of patients, sometimes spanning a lifetime, was part of a particular approach to health care provision championed by the East in the Cold War. Resonating with the practice of welfare states in the West, the socialist world pursued a vision of public health that included free and accessible health care and an integration of preventative medicine and therapeutics. Despite the meagre resources available, neither patients, nor medical staff had any incentive to move patients from the hospital to their home at an early stage and to transfer responsibility for patients with respiratory paralysis from the medical profession to the family. This approach eventually lead to the medicalisation of respiratory patients’ lives and resulted in shared knowledge and practice in medical care among patients, doctors, nurses and technicians.

Living with machines for an extended time, patients gained an intimate knowledge of how their individual machine operated. Some began to intervene into their own care at a very early age. Mária Barabás remembers that by the time she first arrived at the hospital in Debrecen, she could hardly breathe and was turning blue from lack of oxygen. Even though she was quickly placed in an iron lung in Debrecen, she still didn’t get better. ‘Doctors and technicians were busy around me and I still kept fainting, because I was not getting enough air. What could it be, what could it be, they kept trying to find out, when I spoke, me, the 3-year-old, first time in an iron lung, first time having polio, and said, “The pressure is too little,” meaning that there was not enough pressure to push my chest. Later, when my parents were able to laugh again, it became a sort of saying in my family.’^62^

Along with nurses, children would take part in handling emergency situations, such as power outages.’“When everything stopped working, in a power outage, for example, everyone who was able to move even a little bit, was taught to hurry to those who couldn’t even make it for two minutes [without the machine]. … Even at night, we would wake up immediately if it got quiet, and would start screaming for the nurses at once [that the machines are not working]. This reaction became so much a part of me that when years later I moved into my own apartment and there was a power outage, I would drop everything, get into my wheelchair and start hurrying. Only after a metre would I realise that there was no need to run. .^.^. They taught us the responsibility for each other.’^63^

In the case of polio involving respiratory paralysis, nurses became key players in medical care and fulfilled a role of similar significance to physical therapists:

An appropriate nursing staff is of utmost importance for the department. Only specially trained nurses who possess proper knowledge and ability to handle unexpected emergencies may be employed for this work. They have to be capable of operating safely manual respirators, which are in readiness at every patient’s bed, and to perform discharge-suction during the use of positive pressure respirators, every hour or 30 minutes, or in severe cases, if necessary, at 5 to 10 minute intervals. The nurses are responsible for timely removal of the humidity precipitated in the tubes. They must be adequately skilled to assist at interventions. In our department they have to check up the patients’ basic vital signs. … Keeping of a precise record with daily four entries summarizing the machine’s function and numerical data is also among nurses’ duties. Such gravely affected, frequently unconscious, entirely immobile patients require the possibly greatest and most efficient care and nursing.^64^

As on polio wards elsewhere in the world, nurses needed to be prepared for any occasional power outage, had to know how the mechanics of the machines worked, had to be able to operate all respiratory devices, mucus-suction appliances and hand pumps.^65^ This was, in fact, an important part of respiratory treatment for every member of the staff: the medical textbook on respiration therapy from 1963 emphasized that all doctors needed to know intimately how each machine they use works, for that is the only way they can treat complications due to malfunction.^66^

Patients using devices that involved tracheotomy needed to have their trachea suctioned every two hours at the most, since any excess mucus could lead to infection or could block the airways.^67^ Nurses were often required to be ready to leave anything they were doing to perform emergency suction when a patient’s airway became obstructed. Additionally, constant hand washing was needed because of the presence of antibiotic resistant bacteria. The frequent use of disinfectants in turn caused allergic reactions on the skin of many nurses.^68^

The manifold tasks of nurses and the highly specialised skills required of them were only one part of their job. Work on the respiratory wards was very demanding, both physically and emotionally. Not only did the children require intensive attention and highly specialised knowledge, and not only could any situation turn into a critical and life-threatening one, but the children would not eventually get better. They would keep on living there, their care posing the same demands every day and they would never leave the hospital healed. Many nurses, especially those at the beginning of their careers, would soon seek more readily rewarding areas of work.^69^

Polio care, in general, caused a relatively high turnover among nurses, which caused frustration for some of the children, especially ones who spent years in hospital.^70^ One former patient wrote in her memoir about the dread of coming back to the hospital after family leave, surrounded by new children and a completely new staff. Some nurses were sadly missed, while the departure of others—a nurse she referred to as The Witch, who stole children’s food and mistreated the patients—was welcome.^71^ The long work hours, emergency situations and a constant lack of sufficient staff disrupted the life of nurses as well.

The operation of respiratory machines demanded sophisticated knowledge not only in technical terms: anyone operating such devices needed to know about the physiology of respiration itself. It was very important that both the inhalation and exhalation sequences were optimal, since the task of breathing is twofold: first of all, to deliver oxygen to the blood, and second, to remove carbon dioxide from the body. The realisation of how important both processes were in administering assisted breathing was relatively new. As Carl-Gunnar Engström, a Swedish physician and developer of a type of respirator, pointed out in an article in 1953, the mortality rates of patients with respiratory paralysis were still high in 1950, despite mechanical respiration, because of carbon dioxide retention. It was the realisation of the importance of the exhalation sequence and its assistance with newly developed respiratory machines that significantly improved survival rates starting in 1952.^72^ Therefore it was very important that staff could keep an eye on the pressure of the machines and any symptoms of the patients that could reveal an insufficient breathing process.

Similarly to physical therapists, then, nurses transcended conventional divisions of labour and fields of expertise in polio care. Their work fit into the ways in which the demands of the disease’s treatment overthrew hierarchies, involved children in medical decision making and created an unlikely microcosm of real and imagined socialism.

Hospital staff, parents and children explicitly engaging with, or drawing on political ideology was particular to the locality and temporality of a global epidemic. The new experience of severe polio outbreaks in Hungary coincided with the establishment of a new, socialist society. After a devastating war, with social structures, political systems and state organization in flux and drawing on the urgency of a debilitating disease, hospitals became sites in which social and professional roles could be reinterpreted and reshaped. Physical therapists, nurses, and even hospital directors took advantage of the nascent regime in shaping their careers and profession—whether they subscribed to the political system, or not.

The particular care that polio demanded from hospital staff, parents and children also played a significant role in creating opportunities for changing existing power structures. In a way, this aspect of hospital care was less unique to the political system in Hungary. Some of the changes described in this article, like the rise of the field of physical therapy, or the pursuit of alternative treatment options, can be connected more to polio care in general and it occurred in other societies from the Americas to Western Europe. However, the challenges that polio posed—long-term medical care for disabled children—and the solutions sought for those challenges, resonated with political discourses of gender equality and the breakdown of class barriers of socialism. Moreover, a new system of public health management and socialist ideas of universal health care provided patients with extended hospital stays and shaped medical practices, if leaving patients, families and institutions to operate with meagre resources.

The disease, and the urgency of polio epidemics that mapped on to social and political transformation in this Eastern European country, created a space where failed attempts at a new socialist system could flourish. Overthrowing bourgeois hierarchies within the medical profession, creating euqal opportunities for women in work, and prioritising children’s development became realities in the microcosm of the polio hospital while remaining a mere rhetoric outside it. At the same time everyday life and medical practice in the polio hospital demonstrated the limits of this socialist utopia: women were indeed faced with the double burden of professional and domestic work; a lack of equipment and funds restricted treatment options and patients’ lives; and some dictatiorial methods of coercing care or monitoring staff showed the darkest side of political realities in the 1950s.

Life in Hungarian polio hospitals, then, provide us with a richer understanding of both the history of polio care and of the Cold War East. The Eastern European experience of the disease shows the different (and similar) challenges and opportunities this global epidemic raised in a socialist locality. In turn, polio care exposes a different face of Cold War politics in the East: one that shows an active engagement with socialist ideals and space for negotiation, contestation and the pursuit of personal and professional interests.

## Footnotes

1 Dr Dékány Pálné Enyedi Judit, interview by Dora Vargha. Budapest, 11 January 2008.

2 For more on the history of poliomyelitis in general, see David M. Oshinsky, *Polio : An American Story* (Oxford; New York: Oxford University Press, 2005); John R. Paul, *A History of Poliomyelitis*, Yale Studies in the History of Science and Medicine (New Haven, CT: Yale University Press, 1971). Matthew Smallman-Raynor, *Poliomyelitis: Emergence to Eradication*, Oxford Geographical and Environmental Studies (Oxford; New York: Oxford University Press, 2006); Naomi Rogers, *Dirt and Disease: Polio before FDR* (New Brunswick, NJ: Rutgers University Press, 1992).

3 For the Cold War politics of polio vaccines, see Dora Vargha, ‘Between East and West: Polio Vaccination across the Iron Curtain in Cold War Hungary’, *Bulletin of the History of Medicine*, 2014, 88.

4 Elizabeth Kenny, *My Battle and Victory: History of the Discovery of Poliomyelitis as a Systemic Disease* (London: R. Hale, 1955).

5 Naomi Rogers, *Polio Wars. Sister Kenny and the Golden Age of American Medicine* (Oxford: Oxford university Press, 2014); J. Martinez Pérez, R. Ballester Añon, M. I. Porras Gallo and M. J. Báguena, ‘Spanish Health Services and Polio Epidemics in the 20th Century. The “Discovery” of a New Group of Disabled People (1920–1980)’, in Anne Borsay and Pamela Dale, eds, *Disabled Children: Contested Caring, 1850–1979* (London: Pickering and Chatto, 2012); Dilene Raimundo Nascimento, ed., *A Historia Da Poliomielite E De Sua Erradicação No Brasil* (Rio de Janeiro: Casa de Oswaldo Cruz, 2004).

6 Hospital experiences are reconstructed through the internal hospital newspaper, memoirs, journal articles and oral history interviews. The interviews were conducted by the author in the years between 2008 and 2012 in person and via the telephone with former polio patients about their childhood in the 1950s and 1960s, and retired health workers who were involved in polio care in the same decades. All interview subjects consented to the interview being recorded and their name being used in publication of the research. The oral history research was assessed by the IRB administration at Rutgers University. Further interviews recorded by Ádám Csillag for his documentary series, *Gyermekbénulás I–III*, were used. ‘Gyermekbénulás’ directed by Ádám Csillag (Hungary: Csillag és Ádám Film; Fórum Film, 1995).

7 Rezső Hargitai and Ákosné Kiss, eds, *A Gyermekbénulás Elleni Küzdelem: Beszámoló Egy Ma Már Múlttá Váló Rettegett Betegség Ellen Folytatott Hősies Küzdelemről És Felszámolásának Lehetőségéről: A Szent László Kórház Centenáriumára Készült Összeállítás* (Budapest: Literatura Medica, 1994).

8 Acting on the recommendation of Dr János Bókay, Jr., the ministry of interior affairs ordered that all poliomyelitis cases be reported. However, as the order took little effect, the regulation was reinforced after the epidemic of 1926. Exact data on poliomyelitis in Hungary is only available from 1931 based on the work of Dr Aladár Petrilla. Hargitai and Kiss, *A Gyermekbénulás Elleni Küzdelem*, 14. Before the Second World War, poliomyelitis epidemics appeared usually every four years. Outbreaks became more frequent and more deadly from 1952 and were perceived as a constant threat until 1959. The disease took its toll mainly in the summer months, as epidemics hit Hungary in 1952, 1954, 1956, 1957 and 1959. The numbers were grave: the cases of polio rose to 23.8 and 18.3 per 100,000 in the peak years of 1957 and 1959, respectively. (István Dömök: A hazai járványügyi helyzet az élő poliovirus vakcina bevezetése előtt. In: R. Hargitai, ‘Adatok a Gyermekbénulás Leküzdésének Hazai Történetéből’, *Orvosi Hetilap*, 1994, 135, 24–41.) In an international comparison, these numbers are rather high. In England and Wales and West Germany, the highest incidence rate was around 18 per 100,000 in 1950 and 1952, respectively, while it reached nearly 21 in the Netherlands in 1956. (Ulrike Lindner and Stuart, Blume ‘Vaccine Innovation and Adoption: Polio Vaccines in the UK, the Netherlands and West Germany, 1955–1965’, *Medical History*, 2006, 50, Figure 1.)

9 On the United States, see David M. Oshinsky, *Polio: An American Story* (Oxford; New York: Oxford University Press, 2005), 162. On patient numbers in Hungary, see Ottó Rudnai, *The 1959 Poliomyelitis Epidemic in Hungary* Acta Microbiologica (Academiae Scientiarum Hungaricae, 1960).

10 See for instance Peter Kenez, ‘The Hungarian Communist Party and the Catholic Church 1945–1948’, *The Journal of Modern History*, 2003, 75; Katherine Lebow, ‘Public Works, Private Lives: Youth Brigades in Nowa Huta in the 1950s’, *Contemporary European History*, 2001, 10.

11 For more on children in the postwar era and in communist societies, see Catriona Kelly, *Children’s World: Growing up in Russia 1890–1991* (New Haven: Yale University Press, 2007); Izabela Szczepaniak-Wiecha, Agnieszka Malek and Krystyna Slany, ‘The System of Care for Abandoned Children in Poland 1900–1960‘ in Kurt Schilde and Dagmar Schulte, eds, *Need and Care. Glimpses into the Beginnings of Eastern Europe’s Professional Welfare* (Opladen and Farminton Hills: Barbara Budrich Publishers, 2005); Tara Zahra, *The Lost Children. Reconstructing Europe’s Families after World War II* (Cambridge, MA: Harvard University Press, 2011).

12 See Henry Sigerist, *Socialized Medicine in the Soviet Union* (New York: W. W. Norton and Company, 1937); Richard E. Weinerman, and Shirley B. Weinerman, *Social Medicine in Eastern Europe. The Organization of Health Services and the Education of Medical Personnel in Czechoslovakia, Hungary, and Poland* (Cambridge, MA: Harvard University Press, 1969); Bradley Matthys Moore, ‘For the People’s Health: Ideology, Medical Authority and Hygienic Science in Communist Czechoslovakia’, *Social History of Medicine*, 2014, 27, 122–43.

13 The first iron lung arrived in Hungary in 1948, with the cooperation of the American embassy and Andor Bossányi, director of the László Hospital. In the first half of the 1950s, iron lungs began to be produced in Czechoslovakia and GDR, and finally, in the mid-1950s in Hungary as well. By 1959, over 100 Hungarian iron lungs were in use in the country. Domokos Boda, *Sorsfordulók* (Budapest: Harmat, 2004). 60.

14 Tünde Hargitai, telephone interview by Dora Vargha. London, 19 April 2012.

15 Andor Bándi, ‘Heveny Fertőző Betegek Intézeti Elhelyezése’, *Népegészségügy*,1957, no. 10–11.

16 On X-rays and lumbar punctures, see Debreceni Orvostudományi Egyetem, ‘Clinical Discharge Summary’ (Debrecen: Personal archives of György Vargha, 1954). On the effect of poilio on the nervous system, see Dr György Vargha, Iiterview by Dora Vargha. Debrecen, 15 June 2008.

17 Sister Kenny’s methods first arrived in Czechoslovakia, where the medical profession endorsed it from 1950 onwards. Kenny’s treatment concept made it to Hungary through Domokos Boda pediatrician, who translated her manual from Czech. Domokos Boda, interview (Szeged 2009). For more on Kenny in Czechoslovakia, see Naomi Rogers, *Polio Wars. Sister Kenny and the Golden Age of American Medicine* (Oxford: Oxford University Press, 2014).

18 Katalin Parádi, interview by Dora Vargha. Budapest, 27 January 2010.

19 ‘Poliomyelitis’. Papers and Discussions Presented at the Third International Poliomyelitis Conference (paper presented at the International Poliomyelitis Congress, Rome, 1954); ‘Poliomyelitis’. Papers Presented at the Fourth International Poliomyelitis Conference (paper presented at the Fourth International Poliomyelitis Conference, Geneva, 1957); ‘Fifth International Poliomyelitis Conference’, *British Medical Journal*, 1960, 2.

20 Béla Kun, *A Fiatalkorúak Támogatására Hivatott Jótékonycélú Intézmények Magyarországon* (Budapest: Wodianer F. és fiai könyvnyomdai műintézete, 1911).

21 László Lukács, ‘A Budai Gyermekkórház Történetének Periódusai 1956. 12 November–31 December 1963’, in Péter Ferenc, ed., *Gyermekbeteg-ellátás a Rózsadombon 1956–2006* (Budapest: Tudomány Kiadó, 2006). Anna Szívós, *A Heine Medines Mozgászavarok Utókezelése*, ed. Mária Munkácsi, Egészségügyi Felvilágosítás (Budapest: Medicina Egészségügyi Könyvkiadó, 1958).

22 Julianna Bedő, Tamás Kertész and Tibor Szabó, interview by Dora Vargha. Hévíz, 5 November 2010; Parádi, interview; Vargha, interview.

23 Pál Kelemen, telephone interview by Dora Vargha. Berlin, 12 April 2012.

24 Goffman describes total institutions as a ‘place of residence and work where a large number of like-situated individuals, cut off from the wider society for an appreciable period of time, together lead an enclosed, formally administered round of lives’. One of Goffman’s crucial points is that in total institutions, there is a ‘split between a large, managed group, conveniently called inmates, and a small supervisory staff’. Erving Goffman, *Asylums. Essays on the Social Situation of Mental Patients and Other Inmates* (Chicago: Aldine Publishing Company, 1961).

25 For the history of the daily management of iron lung wards, see Lynne M. Dunphy, ‘“The Steel Cocoon”: Tales of the Nurses and Patients of the Iron Lung, 1929–1955’, in Patricia D’Antonio, *et al*., eds, *Nurses’ Work: Issues across Time and Place* (New York: Springer Publishing Company, 2007); Anna László, *Vaspólya* (Budapest: Szépirodalmi könyvkiadó, 1979). For personal accounts of iron lung wards in Hungary and elsewhere, see Franciska Kormos, *Magánkeringő* (Budapest: Aposztróf Kiadó, 2010); Regina Woods, *Tales from Inside the Iron Lung (and How I Got out of It)* (Philadelphia: University of Pennsylvania Press, 1994); Lawrence Alexander, *The Iron Cradle. My Fight against Polio* (London: Hodder and Stoughton, 1955).

26 Lukács explains his choice of the city government as the authority above the hospital, partly by the presence of a colleague there, who was ‘known for his religiousness and anti-marxist views among his friends’. László Lukács, ‘Feljegyzés a Fővárosi Heine-Medin Kórház És Rendelőintézet Alapításáról, Működéséről, Eredményeiről És Ezzel Kapcsolatos Tevékenységéről’, (Budapest: Personal archives of Dr. Prof. Ferenc Péter, 1993), 2.

27 Several figures in the history of polio in Hungary construct their narratives with the inclusion of their religious views and acts. Domokos Boda, head of the polio ward in László hospital, who invented special respiratory equipment and took part in the Sabin vaccination process, opens his autobiography entitled *Twists of Fate* with a quote from the Book of Ecclesiastes and closes it with a psalm; Boda, *Sorsfordulók*. Sándor Koch, leading virologist and personal friend of Albert Sabin goes further to position the narrative of his personal and professional life in terms of religion in the interview book ‘God exists, man happens’. Károly Mezei, *… Isten Van, Az Ember Történik.’ Koch Sándor Virológussal Beszélget Mezei Károly*, Miért Hiszek? (Budapest: Kairosz Kiadó, 2006).

28 Statistics show that in 1963, 38% of the women doctors were under the age of 30 and over 70% were under 40 years old. At the same time, only 17% of male doctors were under 30 and 45% were under 40 years old. This suggests a boom in women entering the profession from the 1950s although their number was significantly below that of men among practising doctors (of the total number of doctors in 1963, 23.5% were women). Központi Statisztikai Hivatal, ‘Egészségügyi Helyzet 1963’, *Statisztikai időszaki közlemények*, 1964, 5, 45.

29 Mária Kovács, *Liberal Professions and Illiberal Politics: Hungary from the Habsburgs to the Holocaust* (Washington: Woodrow Wilson Center Press, 1994).

30 On the need to be fully informed about the medical history of the children, see Elvira Mészáros, interview by Dora Vargha. Budapest, 11 January 2008 on the day-to-day tasks, see Ákosné Dr Kiss, ‘Tartós Gépi Lélegeztetéssel Életben Tartott Postpoliós Légzésbénultak Sorsa’ (Semmelweis University, 1989); Mészáros, interview; László, *Vaspólya*.

31 See *Heine Medin Híradó* (Budapest: Heine Medin Utókezelő Kórház, 1959–1963) for details of staff living on-site while Mészáros, when interviewed, mentioned staff bring their own children to the hospital.

32 Dr Dékány Pálné Enyedi Judit, interview.

33
*Heine Medin Híradó*.

34 Erzsébet Szöllősiné Földesi, interview by Dora Vargha. Budapest, 26 April 2010.

35 Kelemen, telephone interview.

36 Dr Vadkerty Lajosné, ‘Eljött a Nap …, *Heine-Medin Híradó*, 1959, 1.

37 Daniel Wilson points to a similar lack of universality in the experience of polio in the United States. Daniel J. Wilson, *Living with Polio: The Epidemic and Its Survivors* (Chicago: University of Chicago Press, 2005).

38 Cynthia Comacchio, Janet Golden and George Weisz, eds, *Healing the World’s Children: Interdisciplinary Perspectives on Child Health in the Twentieth Century* (Montreal: McGill-Queen’s/Associated Medical Services Studies in the History of Medicine, Health and Society 2008), 8.

39 For children’s experience of polio care in a Western context, see J. Martinez Pérez *et al*., ‘Spanish Health Services and Polio Epidemics in the 20th Century’, 131–43; Heather Green Wooten, *The Polio Years in Texas: Battling the Terrifying Unknown* (Austin: Texas A&M University Press, 2009); Jacqueline Foertsch, *Bracing Accounts: The Literature and Culture of Polio in Postwar America* (Madison: Fairleigh Dickinson University Press, 2008); Marc Shell, *Polio and Its Aftermath: The Paralysis of Culture* (Cambridge, MA: Harvard University Press, 2005).

40 Karen Dubinsky, ‘Children, Ideology and Iconography: How Babies Rule the World’, *Journal of the History of Childhood and Youth*, 2012, 5; Cynthia Comacchio, Janet Golden and George Weisz, eds, *Healing the World’s Children: Interdisciplinary Perspectives on Child Health in the Twentieth Century* (Montreal: McGill-Queen’s/Associated Medical Services Studies in the History of Medicine, Health and Society 2008); Catriona Kelly, ‘Defending Children’s Rights, “in Defense of Peace”. Children and Soviet Cultural Policy’, *Kritika: Explorations in Russian and Eurasian History*, 2008, 9, 711–46.

41 Vargha, interview.

42 Mária Tarnóczy, ‘Fontosabb Feladataink a Heine-Medin-Betegek Rehabilitációjának Megoldása Terén’, *Rheumatológia, Balneológia, Allergológia*, 1963, no. 2.

43 Amy L. Fairchild, ‘The Polio Narratives: Dialogues with FDR’, *The Bulletin of the History of Medicine*, 2001, 75(); J. K. Silver, and Daniel J. Wilson, *Polio Voices: An Oral History from the American Polio Epidemics and Worldwide Eradication Efforts*. The Praeger Series on Contemporary Health and Living (Westport, CT: Praeger, 2007); David Serlin, ‘The Other Arms Race’, in Lennard J. Davis, ed., *The Disability Studies Reader* (New York: Routledge, 2006).

44
*Heine-Medin Híradó* (1959–1962).

45 Vargha, interview.

46 Tarnóczy, ‘Fontosabb Feladataink a Heine-Medin-Betegek Rehabilitációjának Megoldása Terén’.

47 Mária Tarnóczy, ‘Fontosabb Feladataink a Heine-Medin-Betegek Rehabilitációjának Megoldása Terén’, *Rheumatológia, Balneológia, Allergológia*, 1963, no. 2.

48 Dr Mónika Horváth, ‘A Gyógytornász Képzés Története’, Semmelweis University, http://www.sote.hu/intezetek/info/?inst_id=82&page_id=1.

49 Developed by Andras Pető in 1945, Conductive Education integrates medical approaches with educational methods to enable individuals with motor challenges such as spina bifida or celebral palsy to learn how to gain control over their movements. See Judit Forrai, ‘History of a Special Healing Method for Motor-Disordered Chidren: Conductive Education’, *Communicationes De Historia Artis Medicinae*, 2007, 200–201; David Brydan, ‘To Hungary with Love’, in *The Reluctant Internationalists*, blog edited by Jessica Reinisch (London: Birkbeck, University of London, 2014). http://www.bbk.ac.uk/reluctantinternationalists/blog/hungary-love/

50 Lukács, ‘A Budai Gyermekkórház Történetének Periódusai. 12 November  1956–31 December 1963’.

51 Dékány Pálné, interview; *Heine Medin Híradó*.

52 On the need for physical therapist after the war, see Beth Linker, ‘Strength and Science: Gender, Physiotherapy and Medicine in the United States, 1918–35’, *Journal of Women’s History*, 2005, 17; Marilyn Moffat, ‘The History of Physical Therapy Practice in the United States’, *Journal of Physical Therapy Education*, 2003, 17). The situation in Brazil is outlined in Fabio Batalha Monteiro de Barros, ‘Poliomielite, Filantropia E Fisioterapia: O Nascimento Da Profissão De Fisioterapeuta No Rio De Janeiro Dos Anos 1950’, *Ciência & Saúde Coletiva*, 2008, 13. For the United States, see Donald A. Neumann, ‘Polio: Its Impact on the People of the United States and the Emerging Profession of Physical Therapy’, *Journal of Orthopaedic & Sports Physical Therapy*, 2004, 34 and for Spain A. Maturana *et al*., ‘Influencia De Las Epidemias De Poliomielitis Sobre La Rehabilitación En Espana (1949–1969)’, *Rehabilitación*, 2002, 36.

53
*Heine Medin Híradó*. Their relationsips with the nursing staff is mentioned by Mészáros, when interviewed.

54 Out of the 52 physical therapists working in the Heine-Medin hospital, only one or two were men, according to head physical therapist, Dr Dékány Pálné; Judit Dékány Pálné, interview.

55
*Ibid*.

56 Ágnes Soós, interview by Dora Vargha. Budapest, 7 April 2010.

57 On women and socialism, see Agnieszka Zembrzuska, ‘The Socialist Model of Woman in Poland and Its Soviet Prototype’, in Dorothy Rogers *et al*. eds, *Topics in Feminism, History and Philosophy, IWM Junior Visiting Fellows Conferences* (Vienna: IWM, 2000); Jasmina Lukić, Joanna Regulska and Darja Zaviršek, *Women and Citizenship in Central and Eastern Europe* (Aldershot: Ashgate, 2006); Thomas G. Schrand, ‘The Five-Year Plan for Women’s Labour: Constructing Socialism and the “Double Burden”, 1930–1932’, *Europe-Asia Studies*, 1999, 51. Some of the recent scholarhsip on this topic includes See Shana Penn and Jill Massino, *Gender Politics and Everyday Life in State Socialist Eastern and Central Europe* (New York: Palgrave Macmillan, 2009); Izabella Agárdi, ‘The Work of Nostalgia: Women Remembering the Double Burden in Post-Socialist Rural Hungary’, in Gro Hagemann, ed., *Reciprocity and Redistribution : Work and Welfare Reconsidered* (Pisa: Plus-Pisa University Press, 2007).

58 ‘Anya És Gyermekorvos’, *Népakarat*, 8 March 1957.

59 Pósa Dezsőné interviewed by Ádám Csillag in *Gyermekbénulás I*.

60 Dr Kiss, ‘Tartós Gépi Lélegeztetéssel Életben Tartott Postpoliós Légzésbénultak Sorsa’.

61 Ilona Székely, ‘Ház a Baba Utcában’, *A mi kórházunk*, 2009, June.

62 Mária Barabás interviewed by Ádám Csillag in *Gyermekbénulás I*.

63
*Ibid*.

64 Domokos Boda, ‘Evaluation of Recent Methods for the Management of Respiratory Disorders in Poliomyelitis and Other Indications of Mechanical Artificial Respiration’, *Therapia Hungarica*, 1960, 8, 5.

65 Dunphy, ‘“The Steel Cocoon”’.

66 Domokos Boda and László Murányi, *Respiratiós Therapia* (Budapest: Medicina Könyvkiadó, 1963).

67 On the timings for suction, see *Ibid*., 120.

68 Dr Kiss, ‘Tartós Gépi Lélegeztetéssel Életben Tartott Postpoliós Légzésbénultak Sorsa’, 21.

69 Mészáros, interview.

70 For the turnover among nurses, see *Ibid*.

71 Kormos, Magánkeringő.

72 Carl-Gunnar Engström, ‘Treatment of Severe Cases of Respiratory Paralysis by the Engström Universal Respirator’, *British Medical Journal*, 1954, 2.

